# A clinical drug candidate that triggers non-apoptotic cancer cell death

**DOI:** 10.21203/rs.3.rs-4138879/v1

**Published:** 2025-02-11

**Authors:** Scott Dixon, Logan Leak, Ziwei Wang, Weaverly Colleen Lee, Brianna Johnson, Alec Millner, Pin-Joe Ko, Cassandra Decosto, Leslie Magtanong, Joan Ritho, Rachid Skouta, Ekin Atilla-Gokcumen, Chad Myers, Jason Moffat, Charles Boone, Steven Bensinger, Everett Moding, Alby Joseph, Alyssa Chan, Shweta Chitkara, Jenny Salinas, David Nathanson

**Affiliations:** Stanford University; Stanford University; Stanford University School of Medicine; Stanford University; Stanford University; University at Buffalo, SUNY; Stanford University; Stanford University; Stanford University; Stanford University; University of Massachusetts; University at Buffalo, SUNY; University of Minnesota-Twin Cities; University of Toronto; University of California Los Angeles; Stanford University; Stanford University; Stanford University; University at Buffalo; University of Ccalifornia Los Angeles; University of Ccalifornia Los Angeles

**Keywords:** cancer, TECR, palmitate, necrosis, cancer

## Abstract

Small molecules that induce non-apoptotic cell death are of fundamental mechanistic interest and may be useful to treat certain cancers. Here, we report that tegavivint, a drug candidate undergoing human clinical trials, can activate a unique mechanism of non-apoptotic cell death in sarcomas and other cancer cells. This lethal mechanism is distinct from ferroptosis, necroptosis and pyroptosis and requires the lipid metabolic enzyme *trans*-2,3-enoyl-CoA reductase (TECR). TECR is canonically involved in the synthesis of very long chain fatty acids but appears to promote non-apoptotic cell death in response to CIL56 and tegavivint via the synthesis of the saturated long-chain fatty acid palmitate. These findings outline a lipid-dependent non-apoptotic cell death mechanism that can be induced by a drug candidate currently being tested in humans.

## Introduction

Mammalian cells can die via apoptosis or one of several non-apoptotic cell death mechanisms, including pyroptosis, necroptosis and ferroptosis^1, 2^. These non-apoptotic mechanisms are biochemically distinct but converge on the opening of membrane pores or channels that lead to cell swelling and destruction^3–5^. Non-apoptotic cell death processes contribute to both normal and disease biology^6–8^. It may also be possible to treat certain cancers by selectively activating non-apoptotic cell death, especially those that are otherwise therapy-resistant^9–12^. However, the development or identification of drugs that can induce non-apoptotic cancer cell death and be used safely in vivo has been slow.

**Figure 1:**
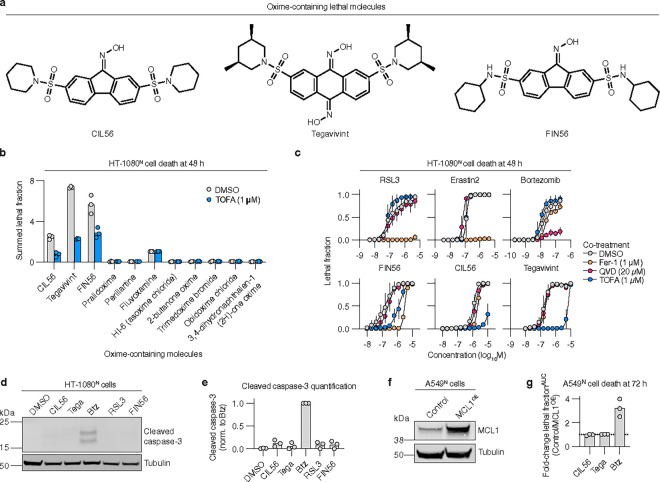
Structurally related synthetic oximes can trigger non-apoptotic cell death. **a**, Small molecule structures. **b**, Cell death determined by imaging of live (nuclear mKate2-positive) and dead (SYTOX Green-positive) cells. Live and dead cell counts were integrated into lethal fraction scores, which were then summed across an 11-point 2-fold dose-response (100 μM high concentration). **c**, Cell death determined by imaging of live and dead cells. A lethal fraction score of 0 = all cells in the population are alive, 1 = all cells in the population are dead. QVD, Q-VD-OPh. **d**,**e**, Protein abundance determined by immunoblotting and quantified across three independent experiments. Compound treatments were EC_90_ values, with samples harvested right before the onset of cell death: CIL56 (1.5 μM, 10 h), tegavivint (Tega, 400 nM, 9 h), bortezomib (40 nM, 13 h), RSL3 (2.4 μM, 2 h), FIN56 (1 μM, 7 h). Representative of three independent blots. **f**, Protein abundance determined by immunoblotting. **g**, Cell death determined by imaging of live and dead cells. Area under the curve (AUC) values were determined from lethal fraction of compound dose-response curves ± MCL1 overexpression and then compared between cell lines. Results in **c** are mean ± SD from three independent experiments. Individual datapoints from separate experiments are shown in **b**, **d** and **g**.

The full spectrum of non-apoptotic cell death mechanisms that can be activated in cancer cells remains unclear. Caspase-independent lethal 56 (CIL56) is an oxime-containing small molecule that kills cancer cells without activating the apoptotic caspases 3 and 7 (Fig. 1a)^13^. CIL56 can trigger a poorly understood form of non-apoptotic cell death that is associated with intracellular protein mis-localization^14, 15^. This mode of cell death is promoted by acetyl-CoA carboxylase 1 (ACC1) and suppressed by the ACC inhibitor 5-(tetradecyloxy)-2-furoic acid (TOFA)^14, 15^. ACC1 synthesizes malonyl-CoA, which is used in the de novo synthesis of palmitate (C16:0) and other fatty acids, implying a link between lipid metabolism and the induction of cell death in response to CIL56. The mechanistic details of the unique CIL56 lethal mechanism remain largely unclear.

Tegavivint (tegatrabetan, BC2059) is a CIL56 analog that has advanced into human clinical trials for several indications, including non-small cell lung cancer (NSCLC), lymphoma, and hepatocellular carcinoma (https://clinicaltrials.gov/expert-search?term=tegavivint). Tegavivint also demonstrates efficacy in mouse models of osteosarcoma, multiple myeloma and leukemia^16–21^, suggesting that this molecule may be useful for the treatment of many cancers. Tegavivint was developed as an inhibitor of Wnt/β-catenin signaling and is reported to induce apoptosis^17, 18, 22, 23^. Here, we present evidence that tegavivint induces non-apoptotic cell death, independent of Wnt/β-catenin pathway inhibition. Tegavivint and CIL56 induce a similar cell death mechanism that is distinct from ferroptosis, necroptosis and pyroptosis, and requires the saturated fatty acid palmitate. Our results suggest that tegavivint could be a useful agent to induce non-apoptotic cancer cell death in diverse settings.

## Results

### Structurally related oximes induce distinct non-apoptotic mechanisms

We asked whether we could identify additional small molecules that triggered cell death in the same manner as CIL56. We tested twelve commercially available drugs and tools compounds that all contained one or more oxime functional group. Each compound was tested for lethality over a 500-fold concentration range, starting at a high dose of 100 μM. We used sensitivity to the ACC inhibitor TOFA to capture our lethal mechanism of interest^14, 15^. HT-1080^N^ fibrosarcoma cells were used as our model. These cells stably express nuclear-localized mKate2 (denoted by a superscript “N”) as a live cell marker^24^ and were incubated in medium containing the dead cell dye SYTOX Green. Cell death was assessed by directly counting live and dead cells using time-lapse imaging^25^. Overall, we found four compounds that were lethal, with the lethality of CIL56, the CIL56 derivative FIN56 (ref.^13^) and the clinical drug candidate tegavivint being reduced by TOFA co-treatment (Fig. 1a,b).

CIL56, tegavivint and FIN56 are structurally related molecules with either one (CIL56, FIN56) or two (tegavivint) oximes and symmetrical hydrocarbon-enriched “wings” (Fig. 1a). Despite this structural similarity, CIL56 is reported to cause an unconventional form of non-apoptotic cell death^13–15^, tegavivint to induce apoptosis^17, 18^, and FIN56 to trigger ferroptosis^13^. These results were obtained from separate studies employing distinct models and methods. We therefore directly compared the lethal mechanisms of CIL56, tegavivint, and FIN56 against one another in the same models. Dying cells have morphologies that can distinguish one form of cell death from another^4^. We therefore first examined the morphology of compound-treated HT-1080^N^ cells using high-resolution time-lapse imaging (**Supplementary Videos 1–6, Extended data Fig. 1a-c**). SYTOX Green was included to assess the onset of membrane permeabilization in relation to observed changes in cell morphology. The proteasome inhibitor bortezomib (Btz) served as a positive control for the induction of apoptosis^26^. The glutathione peroxidase 4 (GPX4) inhibitor 1*S*,3*R*-RSL3 (hereafter, RSL3)^27^ was used as a positive control for ferroptosis induction. Btz-treated cells shrank and accumulated classic apoptotic blebs^28^ by 16 h, with terminal secondary necrosis^5^ apparent in many cells by 24 h. Both RSL3 and FIN56 caused stereotypical ferroptotic cell swelling^3^ as early as 2 h, prior to SYTOX Green entry, which was widespread by 8 h. By contrast, CIL56 and tegavivint did not cause membrane blebbing or cell swelling. Rather, in response to CIL56 or tegavivint the plasma membrane began retracting onto intact and shrinking mKate2-positive nuclei by 8 h, followed 4 to 8 h later by SYTOX Green entry, without widespread secondary necrosis.

These imaging studies suggested that CIL56 and tegavivint triggered a similar cell death phenotype that was distinct from both apoptosis and ferroptosis. We focused first on the apoptosis pathway, which tegavivint is purported to activate^17, 18^. Notably, the pan-caspase inhibitor Q-VD-OPh did not prevent CIL56 or tegavivint from killing HT-1080^N^, SW 872 liposarcoma or SW 982 synovial sarcoma cells (Fig. 1c, **Extended Data** Fig. 1d,e). CIL56 and tegavivint also did not cause the accumulation of cleaved caspase-3 in HT-1080^N^ cells (Fig. 1d,e). Mouse embryonic fibroblasts (MEFs) lacking the key pro-apoptotic proteins *Bax* and *Bak1* (ref.^29^) were resistant to the positive control Btz yet remained sensitive to tegavivint and CIL56 (**Extended Data** Fig. 1f). Furthermore, A549^N^ non-small cell lung carcinoma cells stably overexpressing the anti-apoptotic protein myeloid leukemia 1 (MCL1)^30^ were more resistant than empty vector control cells to Btz but not tegavivint or CIL56 (Fig. 1f,g). Thus, CIL56 and tegavivint both appeared capable of inducing non-apoptotic cell death in these models.

The non-apoptotic cell death phenotype triggered by CIL56 and tegavivint also appeared distinct from ferroptosis and more specifically the phenotype triggered by FIN56. In HT-1080^N^ cells, the ferroptosis-specific inhibitor ferrostatin-1 blocked the lethality of FIN56, RSL3 and erastin2 (ref.^31^) but did not inhibit cell death in response to CIL56 or tegavivint (Fig. 1c). FIN56 causes ferroptosis by inhibiting GPX4 (ref.^13^). We used a functional approach to examine whether CIL56 or tegavivint inhibited GPX4 function. H460 lung cancer cells are normally resistant to GPX4 inhibition due to high expression of a redundant anti-ferroptotic protein, ferroptosis suppressor protein 1 (FSP1)^32^ (**Extended Data** Fig. 2a). In these cells, FSP1 gene disruption (“KO”) unveils a strong dependency upon GPX4 for ferroptosis suppression. This allows for the degree of GPX4 inhibition to be inferred by comparing cell death sensitivity between *FSP1*^*KO*^ and unmodified control cell lines. H460^C^ (expressing mCherry) *FSP1*^*KO*^ cells were strongly sensitized to RSL3 and FIN56 compared to unmodified control cells, with weak sensitization to CIL56 and no sensitization to tegavivint (**Extended Data** Fig. 2b). Likewise, A549^N^ cells treated with the small molecule FSP1 inhibitor^32^ FSEN1 were strongly sensitized to RSL3 and FIN56 but not CIL56 or tegavivint (**Extended Data** Fig. 2c,d). Thus, unlike FIN56, CIL56 and tegavivint did not appear to inhibit GPX4 function or induce ferroptosis in our models.

### Tegavivint and CIL56 do not induce necroptosis or pyroptosis

Our results suggested that CIL56 and tegavivint induced a similar lethal mechanism that was distinct from apoptosis and ferroptosis. We next considered whether this mode of cell death was related to necroptosis or pyroptosis, two well-characterized forms of non-apoptotic cell death^1^. This question seemed pertinent as necroptosis^33^ and pyroptosis^34^ may require the palmitoyltransferase zinc finger DHHC-type palmitoyltransferase 5 (ZDHHC5), an enzyme that we showed can promote cell death in response to CIL56 (ref. ^14^). We confirmed that HT-1080^N^ *ZDHHC5* gene-disrupted (“KO”) cells^14^ were more resistant to CIL56 than unmodified Control cells (**Extended Data** Fig. 3a,b). However, *ZDHHC5*^*KO*^ and Control cells were equally sensitive to tegavivint (**Extended Data** Fig. 3b). Five additional lines of evidence indicated that the lethality of CIL56 and tegavivint in our cancer models was unlikely to be explained by the induction of necroptosis or pyroptosis. First, tegavivint and CIL56 did not cause pronounced cell swelling (**Extended data** Fig. 1a), a characteristic morphological feature of necroptosis and pyroptosis^4^. Second, receptor interacting serine/threonine kinase 3 (*RIPK3*), which is necessary for the execution of necroptosis^4^, is not expressed in HT-1080 cells (**Extended Data** Fig. 3c), and we showed that genetic silencing of *RIPK3* in a necroptosis-sensitive cell line does not prevent cell death in response to CIL56^14^. Third, like other non-myeloid cells, HT-1080 cells are resistant to pyroptosis^35^. Fourth, CIL56 and tegavivint alone are lethal to HT-1080 and other cancer cells without the typical “priming” stimulus required for sensitivity to pyroptosis (e.g., lipopolysaccharide, LPS). Fifth, in a proper LPS-primed THP-1 monocyte model of pyroptosis^36^, the inflammasome inhibitor MCC905 blocked cell death in response to the pyroptotic stimulus nigericin but not in response to tegavivint or CIL56 (**Extended Data** Fig. 3d). Collectively, the induction of necroptosis or pyroptosis seemed unlikely to explain the unique lethal mechanism triggered by CIL56 and tegavivint.

### TECR promotes the lethality of CIL56 and tegavivint

**Figure 2:**
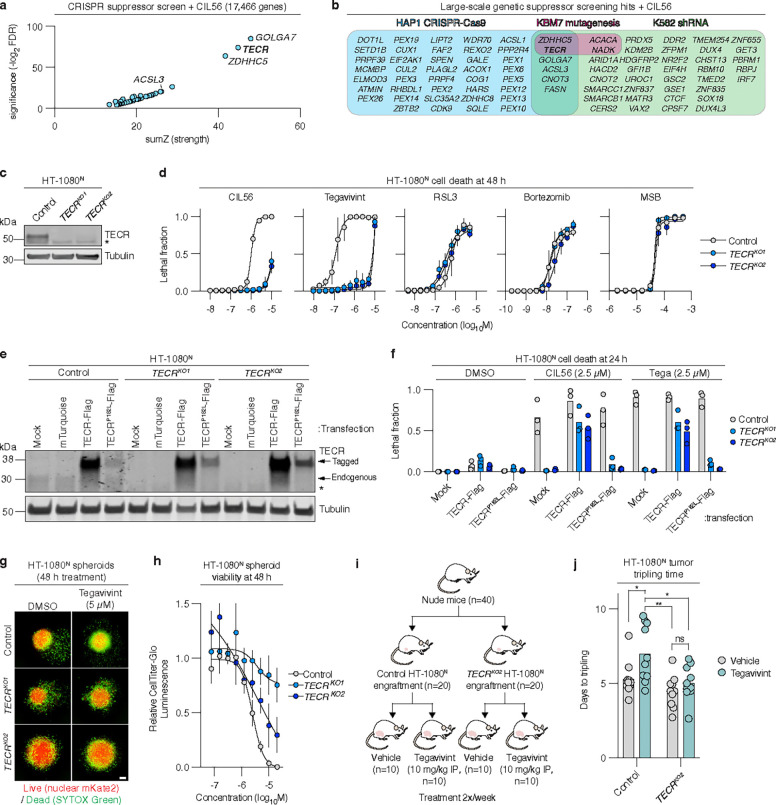
TECR is required for cell death. **a**, Summary of CIL56 suppressor genes identified in a HAP1 cell CRISPR-Cas9 screen. **b**, Overlap of results of different CIL56 genetic suppressor screens. **c**, Protein abundance determined by immunoblotting. *indicates a non-specific band. **d**, Cell death determined by imaging of live and dead cells. A lethal fraction score of 0 = all cells in the population are alive, 1 = all cells in the population are dead. MSB: menadione sodium bisulfite. **e**, Protein abundance determined by immunoblotting. **f**, Cell death determined by imaging following transient transfection. Individual datapoints from three independent experiments are shown. **g**, Images of spheroids grown in ultralow attachment plates then treated as indicated. Images are representative of three independent experiments. **h**, Quantification of spheroid viability using CellTiter-Glo 3D. **i**, Cartoon overview of a mouse xenograft tumor treatment experiment. **j**, Quantification of xenograft tumor tripling time. Each datapoint represents one mouse/one xenograft tumor. Significance was determined using a two-way ANOVA with Tukey’s multiple comparison test (n = 10 xenografts /condition), *P < 0.05, **P< 0.01. Blots in **c** and **e** are representative of three independent experiments. Results in **d** and **h** are mean ± SD from three independent experiments.

**Figure 3:**
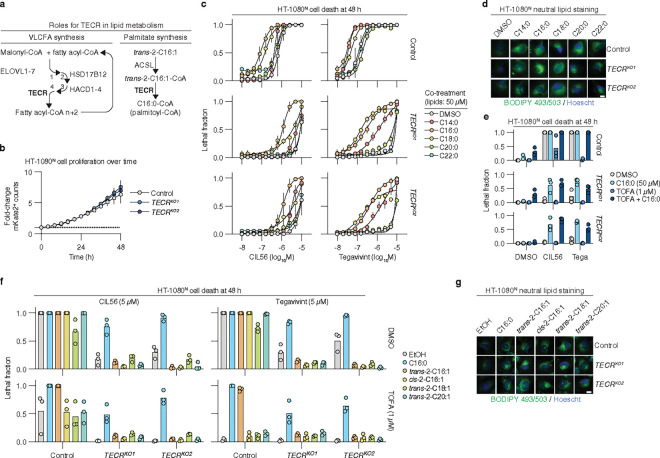
Palmitate is required for non-apoptotic cell death. **a**, Cartoon depicting the roles of TECR in lipid metabolism. The proteins involved in each step are indicated. **b**, Cell proliferation determined by counting mKate2-positive (mKate2^+^) live cells over time. Counts are normalized to t = 0. **c**, Cell death determined by imaging of live and dead cells. A lethal fraction score of 0 = all cells in the population are alive, 1 = all cells in the population are dead. **d**, Neutral lipid staining detected using BODIPY 493/503. Cells were incubated with fatty acids at 25 μM for 6 h. Scale bar = 10 μm. **e**, Cell death determined by imaging. CIL56 and tegavivint were used at 2.5 μM. Individual datapoints from three independent experiments are shown. **f**, Cell death determined by imaging. **g**, Cell death determined by imaging. Fatty acids were used at 25 μM. **h**, Neutral lipid staining detected using BODIPY 493/503. Cells were incubated with fatty acids at 25 μM for 6 h. Scale bar = 10 μm. Images are representative of three experiments. Results in **b**, **c**, and **f** are mean ± SD from three independent experiments. Results in **e** and **g** are individual datapoints from separate experiments.

To gain further insight into the lethal mechanism induced by CIL56 we executed a genome-wide CRISPR-Cas9 suppressor screen in human HAP1 haploid cells. This screen identified 45 genes whose disruption resulted in significant resistance to CIL56 (Benjamini–Hochberg adj. p < 0.05, Fig. 2a,b). We intersected these results with two previous genetic screens^14, 15^. Only two genes were identified in all three genetic suppressor screens: *ZDHHC5* and the lipid metabolic gene trans-2,3-enoyl-CoA reductase (*TECR*) (Fig. 3b). *ZDHHC5* disruption had no effect on tegavivint-induced cell death (**Extended data** Fig. 3a,b). We therefore focused on *TECR*, an understudied gene, with the hypothesis that it was required for cell death in response to both CIL56 and tegavivint.

We isolated clonal HT-1080^N^ *TECR* gene-disrupted (“KO”) cell lines that lacked TECR protein expression, along with a Control cell line that went through the CRISPR process but was unmodified (Fig. 2c). *TECR* gene disruption attenuated cell death and cell death-associated morphological changes caused by CIL56 and tegavivint (**Extended data** Fig. 4a). Overall, *TECR* gene-disrupted cells were ~ 10-fold more resistant to CIL56 and ~ 100-fold more resistant to tegavivint than Control cells with little or no resistance to small molecule inducers of ferroptosis (RSL3, ML162, erastin2), other forms of caspase-independent cell death (CIL41, CIL70)^13^, cuproptosis (elesclomol)^37^, zinc-dependent necrosis (zinc pyrithione)^25^, alkaliptosis (JTC-801)^38^, methuosis (MOMIPP, vaquinol-1)^39^, triaptosis (menadione sodium bisulfite, menadione, VPS34-IN-1)^40^ or apoptosis (Btz, thapsigargin) (Fig. 2d and **Extended data** Fig. 4b,c).

Sensitivity to CIL56 and tegavivint was restored in HT-1080^N^ *TECR* gene-disrupted cells by re-expressing wild-type TECR but not a disease-associated mutant (P182L) that has reduced enzymatic activity^41^ (Fig. 2e,f). The TECR^P182L^ mutant protein is less stable than the wild-type protein^41^ but still expressed in *TECR*^*KO1/2*^ cells at levels equivalent to endogenous TECR in Control cells. In addition to HT-1080^N^ cells, we generated *TECR* gene-disrupted SW 872^N^ and HAP1^N^ cell lines and found them to be more resistant to CIL56 than paired Control cell lines (**Extended data** Fig. 4d-f). Similar to HT-1080^N^ cells, CIL56 sensitivity was also restored in SW872^N^ *TECR*^*KO2*^ cells by re-expressing wild-type TECR but not TECR^P182L^ (**Extended data** Fig. 4g,h).

We next assessed whether the response to tegavivint remained *TECR*-dependent in more complex models. Cancer cells growing in non-adherent conditions can better recapitulate the in vivo tumor environment^42^. HT-1080^N^ *TECR*^*KO1/2*^ cells cultured as spheroids in non-adherent plates were more resistant to tegavivint than Control cells cultured under the same conditions (Fig. 2g,h). Furthermore, HT-1080^N^ *TECR*^*KO2*^ cells implanted in the thigh muscle of nude (*Foxn1*^*nu*^) mice as xenografts were also significantly more resistant than Control cells to tegavivint administration (10 mg/kg bw, i.p., 2x/wk), as reflected in time to tumor tripling (*P* < 0.05, two-way ANOVA with Tukey’s post-hoc tests) (Fig. 2i,j). Thus, *TECR* appeared to promote response to tegavivint both in vitro and in more complex in vivo models.

### Palmitate promotes cell death downstream of TECR

TECR catalyzes the last step in the elongation of long chain fatty acids to very long chain fatty acids (VLCFAs)^41, 43^ (Fig. 3a). VLCFA elongation is non-essential in other cells^43^ and indeed HT-1080^N^ *TECR*^*KO1/2*^ cells proliferated normally, suggesting that resistance to CIL56 and tegavivint was not associated with slower cell division (Fig. 3b). In DepMap^44^, *TECR* gene dependency correlates most strongly with that of *HSD17B12*, encoding the enzyme that catalyzes the second step in long-chain fatty acid elongation (Fig. 3a, **Extended data** Fig. 5a). However, *HSD17B12* was not recovered as a suppressor of CIL56-induced cell death in our genome-wide screens (Fig. 2a). Short interfering RNA (siRNA)-mediated silencing of *HSD17B12* in HT-1080^N^ cells also did not inhibit CIL56-induced cell death (**Extended data** Fig. 5b,c). These data suggested that *TECR* may have an alternative role in enabling CIL56 and tegavivint to kill cancer cells.

Separate from its role in VLCFA synthesis, TECR also specifically synthesizes the long chain fatty acid palmitoyl-CoA as part of a distinct lipid metabolic pathway^45^ (Fig. 3a). We therefore examined the effects of palmitate and other saturated fatty acids on the lethality of CIL56 and tegavivint. The addition of 50 μM palmitate (C16:0) or other long-chain fatty acids (C14:0 and C18:0) sensitized HT-1080^N^ Control cells to CIL56 and/or tegavivint, while the VLCFAs C20:0 and C22:0 had no effect (Fig. 3c). Strikingly, the addition of palmitate to HT-1080^N^ *TECR*^*KO1/2*^ cells also restored the lethality of CIL56 and tegavivint almost to the level of Control cells (Fig. 3c). C14:0 and C18:0 had less potent effects in this assay than palmitate, while VLCFAs had no effect (Fig. 3c). All exogenous lipids appeared to be taken into cells, as indicated by the stimulation of neutral lipid synthesis (Fig. 3d, **Extended data** Fig. 6a). In addition to HT-1080^N^ cells, exogenous palmitate also restored cell death in SW 872^N^ *TECR*^*KO1/2*^ cells and HAP1^N^ *TECR*^*KO1/2*^ cells treated with CIL56 or tegavivint (**Extended data** Fig. 6b). These results were consistent with the notion that palmitate acts downstream of TECR to promote cell death in response to CIL56 or tegavivint.

The addition of palmitate to cultured cells at high concentrations (e.g., 200 μM) can trigger a lethal mechanism referred to as lipoapoptosis^46^. Importantly, the amount of palmitate sufficient to restore CIL56 or tegavivint-induced cell death in *TECR* gene-disrupted cell lines was minimally lethal alone (Fig. 3c). Direct application of 200 μM palmitate killed HT-1080^N^ Control and *TECR*^*KO1/2*^ cell lines to a similar extent, and cell death under these conditions was attenuated by the pan-caspase inhibitor Q-VD-OPh but not by TOFA, consistent with the induction of apoptosis (**Extended data** Fig. 6c). Thus, the ability of palmitate to promote cell death in response to CIL56 or tegavivint appeared distinct from its ability to induce lipoapoptosis.

TECR synthesizes palmitoyl-CoA from the “activated” 16-carbon monounsaturated lipid *trans*-2-hexadecenoyl-CoA (*trans*-C16:1-CoA)^45^, whose direct precursor is *trans*-2-hexadecenoic acid (*trans*-2-C16:1) (Fig. 3a). We reasoned that if TECR promoted death via palmitoyl-CoA synthesis, then addition of the TECR substrate precursor *trans*-2-C16:1 should not be able to restore cell death in *TECR*-mutant cells. To test this hypothesis, we needed a condition that would allow us to first confirm the activity of both palmitate and *trans*-2-C16:1 in a different context where TECR activity was intact. Usefully, the lethality of CIL56 and tegavivint was suppressed by TOFA in HT-1080^N^, HAP1^N^, A549^N^, T98G^N^ glioblastoma and Panc-1^N^ pancreatic cancer cells and restored in all cases by exogenous palmitate (Fig. 3e, **Extended data** Fig. 6b,d). *Trans*-2-C16:1 (50 μM) likewise restored CIL56 or tegavivint-induced cell death in HT-1080^N^ or SW 872^N^ Control cells co-treated with TOFA (Fig. 3f, **Extended data** Fig. 6e). However, unlike palmitate, *trans*-2-C16:1 had no ability to restore cell death in *TECR*^*KO1/2*^ cells (Fig. 3f). The effect of *trans*-2-C16:1 was specific: the structurally related monounsaturated lipids *cis*-2-C16:1, *trans*-2-C18:1, and *trans*-2-C20:1 had no effect on CIL56 or tegavivint-induced cell death in HT-1080^N^ cells despite evidence of cellular uptake (Fig. 3f,g, **Extended data** Fig. 6f). Thus, TECR was required for *trans*-2-C16:1 to promote CIL56 or tegavivint-induced cell death.

*Trans*-2-C16:1 itself is not a substrate for TECR; this metabolite must first be activated to *trans*-2-C16:1-CoA by one or more acyl-CoA synthetase long chain (ACSL) enzymes^45^ (Fig. 3a). We recovered *ACSL3* in two of three CIL56 genetic suppressor screens, and *ACSL1* in the HAP1 CRISPR screen (Fig. 2b). Accordingly, we hypothesized that *ACSL3* and/or *ACSL1* were required for cell death in response to CIL56 and tegavivint. In support of this hypothesis, HEK 293 *ACSL3* gene-disrupted cell lines^47^ showed reduced sensitivity to CIL56 compared to unmodified Control cells (**Extended data** Fig. 6g). Furthermore, in HT-1080^N^ cells, the broad spectrum ACSL inhibitor triacsin C^48^ potently suppressed cell death in response to both CIL56 and tegavivint, and cell death could not be restored by addition of exogenous *trans*-2-C16:1 (**Extended data** Fig. 6h). Thus, long chain fatty acid activation appeared necessary for cell death in response to CIL56 and tegavivint.

### Cell death induction is sensitive to low dose 2-bromopalmitate

**Figure 4:**
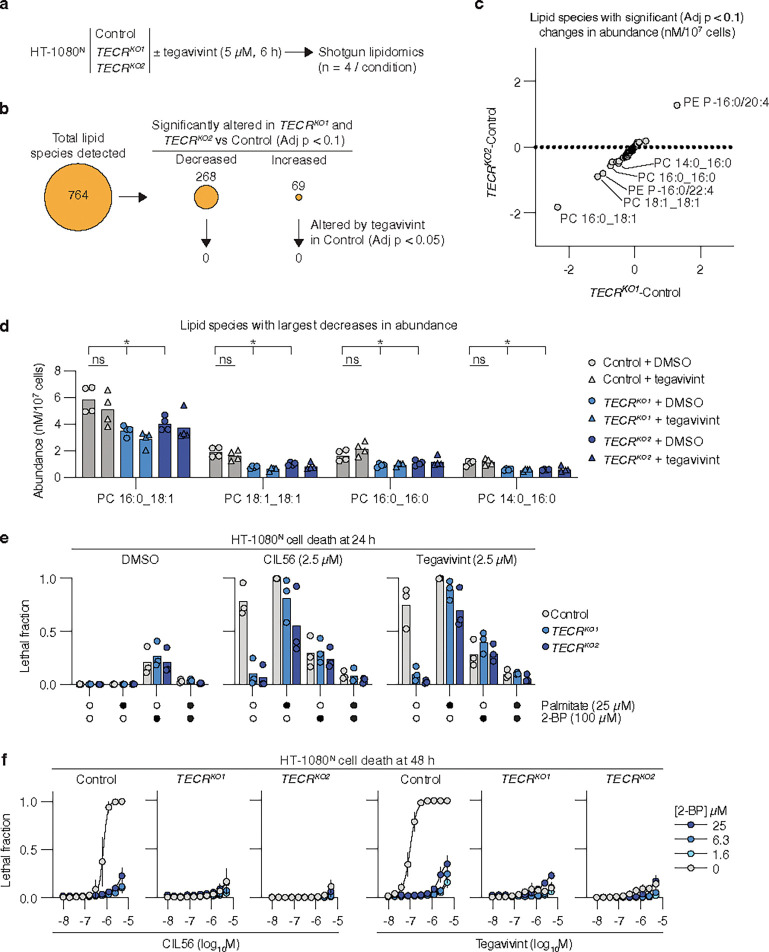
Inhibition of palmitate metabolism blocks cell death. **a**, Schematic of the shotgun lipidomic analysis. **b**, Summary of lipid species detected in the shotgun lipidomic analysis. Individual lipid species that differed between *TECR*^*KO1*^ and *TECR*^*KO2*^ versus the Control cell line were determined using t-tests followed by the Benjamini–Hochberg adjustment for multiple comparisons. Data are from four independent experimental replicates for each condition. **c**, Changes in lipid abundance for all significantly altered lipids identified in **b**. Each datapoint represents one lipid species. **d**, Abundance of individual lipid species from **c**. *indicates the differences were statistically significant, as described for **b**. ns = not significant. **e**, Cell death determined by imaging of live and dead cells. A lethal fraction score of 0 = all cells in the population are alive, 1 = all cells in the population are dead. **f**, Cell death determined by imaging of live and dead cells. Results in **d** and **e** are individual datapoints from separate experiments, while results in **c** are mean ± SD from three independent experiments.

How palmitoyl-CoA promoted cell death downstream of TECR in cells treated with CIL56 or tegavivint therefore remained unclear. One model was that CIL56 and tegavivint caused TECR-dependent changes in lipid metabolism that were lethal to the cell. To investigate, we used shotgun lipidomics^49^ to examine HT-1080^N^ Control and *TECR*^*KO1/2*^ cells treated with tegavivint (2.5 μM, 5 h) or vehicle control (DMSO) (Fig. 4a). In total, 764 lipids were detected across all conditions in at least three of four independent experimental samples. Focusing first on DMSO-treated cells, 44% (337/764) of all detected lipids were significantly altered in *TECR*^*KO1*^ and *TECR*^*KO2*^ cell lines versus the Control cell line (Benjamini–Hochberg adj. p < 0.1). 80% (268/337) of altered lipids were decreased in abundance in *TECR*^*KO1/2*^ cells versus Control cells, including multiple VLCFA-containing ceramides, consistent with the function of TECR in fatty acid elongation^43^ (Fig. 4b, **Extended data Fig. 7**). However, in terms of absolute lipid abundance, the greatest changes in *TECR*^*KO1/2*^ versus Control cells were decreased levels of phosphatidylcholine (PC) 16:0_18:1, PC 18:1_18:1, phosphatidylethanolamine (PE) P-16:0/22:4, PC 16:0_16:0 and PC 14:0_16:0 and increased levels of PE P-16:0/20:4 (Fig. 4c,d). The accumulation of PE P-16:0/20:4 and simultaneous depletion of PE P-16:0/22:4 is likely explained by a failure to elongate C20:4 (from the growth medium) to C22:4 in the absence of TECR. By contrast, the depletions of PC 16:0_18:1, PC 18:1_18:1, PC 16:0_16:0 and PC 14:0_16:0 in *TECR*-disrupted cells were not matched by any corresponding accumulation of alternative species and appeared consistent with disrupted palmitate synthesis. While these data were consistent with an important role for TECR in lipid metabolism in general, and palmitate metabolism specifically, changes in phospholipid metabolism *per se* did not seem to be a feature of tegavivint exposure: none of the *TECR*-dependent lipid species were significantly altered in abundance in Control cells by tegavivint treatment (Benjamini–Hochberg adj. p > 0.05) (Fig. 4b,d).

An alternative model for how palmitoyl-CoA promoted cell death was that this metabolite was required for the activity of one or more death-promoting enzymes, downstream of TECR. To investigate, we employed the palmitate analog 2-bromopalmitate (2-BP). 2-BP can be activated to 2-BP-CoA and then compete with palmitoyl-CoA and act as a promiscuous inhibitor for dozens of enzymes, including those involved in fatty acid oxidation, triglyceride synthesis, and protein palmitoylation^50–54^. 2-BP (100 μM) fully suppressed cell death in response to both CIL56 and tegavivint in HT-1080^N^ Control cells (Fig. 4e). 2-BP also prevented palmitate from restoring cell death in *TECR*^*KO1/2*^ cells treated with CIL56 or tegavivint (Fig. 4e). 2-BP is often used to inhibit protein palmitoylation, where it is used at concentration of 100 μM or more^51, 53, 54^. Remarkably, concentrations of 2-BP under 2 μM were sufficient to completely inhibit cell death in response to both CIL56 and tegavivint in HT-1080^N^ cells (Fig. 4f). This implied that the death-promoting activity of palmitoyl-CoA may be distinct from the inhibition of protein palmitoylation.

### Tegavivint kill cells independent of Wnt/β-catenin pathway inhibition

**Figure 5:**
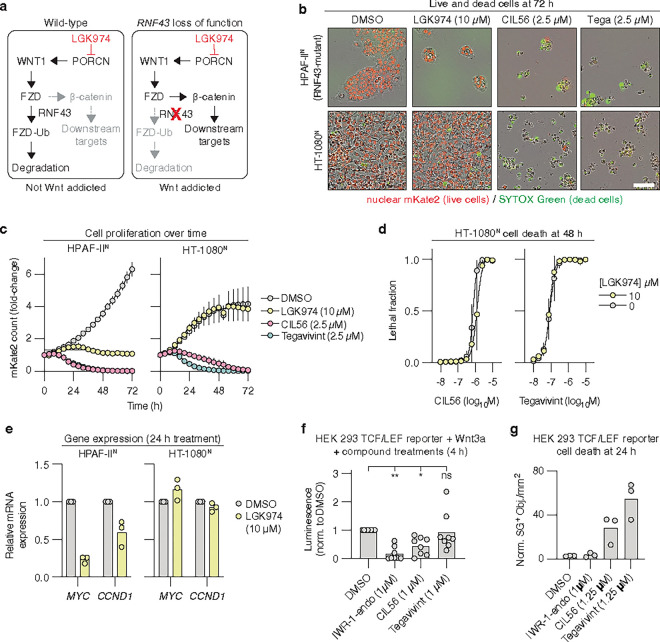
Tegavivint does not kill cells via Wnt/b-catenin pathway inhibition. **a**, Cartoon illustration of the Wnt/b-catenin signaling pathway highlighting the role of RNF43. FZD, frizzled class receptor; Ub, ubiquitin. **b**, Representative images of live and dead cells from one of three independent experiments. Scale bar = 100 μm. **c**, Quantitation of imaging shown in **b**. Fold-change in live cell counts relative to t = 0 determined by counting of mKate2-positive objects over time. Data are mean ± SD from three independent experiments. **e**, Relative mRNA expression determined using reverse transcription coupled to quantitative polymerase chain reaction analysis. Individual datapoints from three independent experiments are shown. **f**, Luciferase signal determined by plate reader and normalized to DMSO condition. Data were analyzed using one-way ANOVA with Tukey’s posthoc tests. **P < 0.01, *P < 0.05, ns = not significant. **g**, Cell death determined by counting of SYTOX Green positive (SG+, i.e., dead) cells. Results in **c** and **d** are mean ± SD from three independent experiments. Results in **e**, **f** and **g** are individual datapoints from independent experiments.

Tegavivint is proposed to cause cell death by inhibiting the Wnt/β-catenin pathway^16, 23^. We asked whether Wnt pathway inhibition explained the ability of tegavivint and CIL56 to cause non-apoptotic cell death. We approached this question functionally, using cancer cell lines known to be dependent upon Wnt pathway activity. *RNF43*-mutant pancreatic cancer cells have stabilized Frizzled (Wnt receptor), resulting in Wnt pathway addiction^55, 56^ (Fig. 5a). We hypothesized that these Wnt-dependent cells should be especially poised to respond to CIL56 and tegavivint. CIL56 and tegavivint were lethal to *RNF43*-mutant^55^ HPAF-II^N^ and Capan-2 cells (Fig. 5b,c, **Extended Data Fig. 8a,b**). By contrast, a *bona fide* Wnt pathway blocker, the porcupine *O*-acyltransferase (PORCN) inhibitor LGK974 (ref.^57^), did not cause cell death and instead arrested the proliferation of these cells, consistent with published data^55–57^ (Fig. 5b,c, **Extended Data Fig. 8a,b**). In HT-1080^N^ cells, where CIL56 and tegavivint are highly lethal, LGK974 did not even inhibit proliferation (Fig. 5b,c, **Extended Data Fig. 8a,b**), perhaps because these cells are not addicted to Wnt pathway activity. Indeed, in HT-1080^N^ cells, the addition of LGK974 did not enhance the lethality of CIL56 or tegavivint, as would be expected if acting on the same mechanism (Fig. 5e).

These results called into question not only a role for Wnt pathway inhibition in the induction of cell death, but whether CIL56 and tegavivint could inhibit Wnt pathway activity at all. Treatment with the positive control LGK974 for 24 h lowered the expression of two β-catenin target genes (*MYC* and *CCND1*) in HPAF-II^N^ and Capan-2 cells but not in HT-1080^N^ cells (Fig. 5d). Treatment with lethal doses of tegavivint or CIL56 for 5 h, before the onset of cell death, did not consistently reduce the expression of either gene in HPAF-II^N^ or HT-1080^N^ cells (**Extended Data Fig. 8d**). Moreover, tegavivint did not consistently prevent the expression of a Wnt-sensitive luciferase reporter in engineered HEK 293 cells^58^ (Fig. 5f). A positive control for this assay, the tankyrase inhibitor IWR-1-endo^59^, did inhibit Wnt-stimulated luciferase expression but unlike tegavivint and CIL56 was not lethal to these cells (Fig. 5f,g). Thus, Wnt pathway inhibition seemed unlikely to explain the ability of tegavivint to induce non-apoptotic cell death in HT-1080 and other cells.

### Tegavivint has broad-spectrum anti-cancer activity

**Figure 6:**
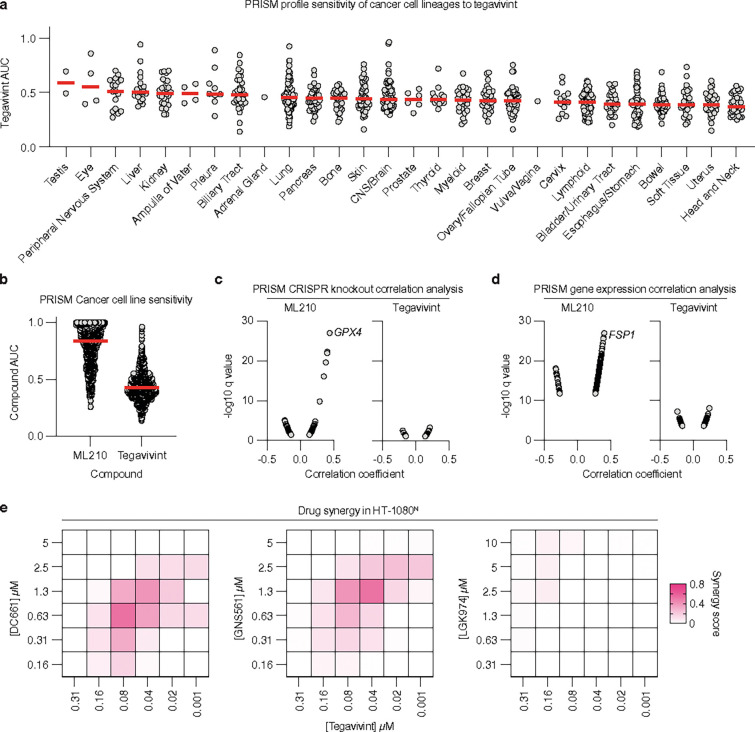
Tegavivint has broad spectrum lethality. **a**, PRISM profiling of cancer cell line sensitivity to tegavivint. Sensitivity is reflected in the area under the curve (AUC) value, with lower values indicated greater sensitivity. Each data point represents one cell line, classified by primary lineage. **b**, Summary of PRISM profiling sensitivities (AUC) to the GPX4 inhibitor ML210 and tegavivint. **c**, Correlation analysis between compound sensitivities and CRISPR knockout profiles. **d**, Correlation analysis between compound sensitivities and gene expression. **e**, Compound synergy analysis. Results in the synergy heatmap represent mean values from three independent experiments.

Our results suggested that tegavivint lethality was unlikely to be limited to cancer cells with hyperactive Wnt pathway activity. To explore the potential scope of tegavivint utility, we profiled the activity of this compound in 864 pooled and barcoded cancer cell lines using the Broad PRISM platform^60, 61^. Tegavivint showed activity towards cancer cell lines from all lineages, with relatively higher median potency towards cancer cell lines from head and neck and uterine lineages and lower activity towards eye, peripheral nervous system and liver lineages (Fig. 6a). However, cell lines within each lineage typically spanned a range of tegavivint sensitivities (Fig. 6a). Only two weak mutational corelates of tegavivint activity were detected (*FOXM1*, *PHF20L1*), neither of which are related to Wnt signaling (**Extended data Fig. 9a**). In fact, tegavivint activity across cancer cell lines did not correlate strongly or informatively with existing CRISPR single gene knockout or gene expression features (Fig. 6b–d). By comparison, activity of the GPX4 inhibitor ML210 was highly correlated with CRISPR-mediated disruption of its known target (GPX4) and the expression of a known resistance gene (*FSP1*)^62, 63^ (Fig. 6b–d). These results indicated that the range of tegavivint activity may be wider than anticipated.

Finally, we tested whether other drug candidates that target palmitate metabolism would interact with tegavivint. DC661 and GNS561 (Ezurpimtrostat) are structurally distinct inhibitors of palmitoyl-protein thioesterase 1 (PPT1), an enzyme that removes palmitate from proteins in the lysosome^64, 65^. DC661 is a pre-clinical compound while, like tegavivint, GNS561 is being tested in human oncology clinical trials^66^. We treated HT-1080^N^ cells with low doses of tegavivint together with either DC661 or GNS561 and observed enhanced lethality when both drug candidates where combined together (Fig. 6e). Similar effects were observed for CIL56 (**Extended data Fig. 9b**). By contrast, little or no synergy was observed between tegavivint or CIL56 and the Wnt pathway inhibitor LGK974 (Fig. 6e, **Extended data Fig. 9c**).

## Discussion

Historically, synthetic small molecules like erastin or RSL3 have been useful tools to discover and characterize new non-apoptotic cell death mechanisms^1, 2^. Some of these mechanisms and lethal small molecules may ultimately be useful for the treatment of cancer. However, this is a lengthy process. For example, despite over a decade of work, there are still no agents that induce ferroptosis undergoing human clinical trials. Part of the concern with agents that induce ferroptosis is the obvious potential for toxicity with inhibition of GPX4 or other essential proteins^67^. Unexpectedly, we find that tegavivint can induce non-apoptotic cell death. Tegavivint already has a well-established safety profile in mice^16–21^ and is now being tested in humans. Apart from its potential clinical utility, tegavivint may provide a useful means to ask general questions about drug-induced non-apoptotic cancer cell death in vivo.

Our evidence indicates that tegavivint and CIL56 can cause a cell death phenotype that is distinct from apoptosis, ferroptosis, necroptosis, and pyroptosis^14, 15^. TECR is required for cell death in response to tegavivint and CIL56, but not other lethal stimuli that trigger apoptosis, ferroptosis, triaptosis^40^, cuproptosis^37^ or methuosis^39^. Nonetheless, there are good reasons to be wary of prematurely affixing a name to this form of cell death. First, unique markers for this mode of cell death (e.g., like the iron-dependent accumulation of lipid peroxides in ferroptosis)^2^ remain to be defined. Second, whether other small molecules or stimuli can induce this lethal mechanism is unknown. Third, the broader physiological relevance of this mechanism is unclear. That said, for ease of exposition here in this Discussion, we refer to this process as lipid-dependent necrosis (LiDN).

LiDN is promoted by TECR. Our lipid supplementation experiments suggest that the function of TECR in palmitoyl-CoA synthesis^45^, rather than its canonical function in fatty acid elongation and VLCFA synthesis, is most important for LiDN. Other lines of evidence, including the rescue of cell death by the TECR substrate *trans*-2-C16:1, by the ACSL inhibitor triacsin C and by the palmitate analog 2-BP, are all consistent with palmitoyl-CoA being sufficient to promote LiDN. That said, it is unclear whether palmitoyl-CoA is necessary: other long chain fatty acids beside palmitate (i.e., C14:0, C18:0) also restored cell death in *TECR*-mutant cells. It is possible that, downstream of TECR, these lipids can substitute for palmitate equally well. Alternatively, these lipids could be metabolized to palmitate before contributing to LiDN.

How palmitoyl-CoA promotes LiDN downstream of TECR remains to be fully worked out. LiDN is blocked by 2-BP, which is commonly used to inhibit protein palmitoylation. We previously showed^14^ that the protein palmitoyltransferase ZDHHC5 promotes cell death in response to CIL56. However, loss of *ZDHHC5* did not inhibit tegavivint-induced cell death. One model is that tegavivint-induced LiDN specifically requires a different ZDHHC enzyme or more than one ZDHHC enzyme operating in parallel, which are targets of 2-bromopalmitate. However, 2-bromopalmitate completely inhibited LiDN at doses (< 2 μM) far below those typically used to inhibit protein palmitoylation (> 50 μM)^51^. 2-BP can directly inhibit enzymes involved in mitochondrial beta-oxidation, as well as glycolysis and cytochrome P450 function^52^. Thus, an alternative model is that LiDN requires one or more of these enzymes.

Tegavivint was developed as an inhibitor of the Wnt/β-catenin pathway and is reported to induce apoptosis^16, 19, 68^. However, many cancer drugs have novel targets and lethal mechanisms of action that are only discovered over time^60, 69, 70^. We find that tegavivint can trigger LiDN in a wide range of cancer cells. Our evidence suggests that Wnt/β-catenin pathway inhibition is unlikely to explain the induction of LiDN by tegavivint. This may not be entirely surprising, as Wnt signaling is thought to promote cancer cell proliferation^55–57^, not survival. Ongoing human clinical trials for cancers that are not generally characterized by Wnt pathway activation, such as NSCLC, lymphoma and hepatocellular carcinoma suggests that broader applications for tegavivint are already anticipated.

Analysis of tegavivint lethality in the PRISM assay did not identify strong correlations between the activity of tegavivint and either CRISPR gene disruption profiles or gene expression. This might suggest that tegavivint acts through the modulation of multiple targets, or possibly through mechanisms that do not involve direct protein engagement. Further investigation of related oxime-based chemical scaffolds may provide interesting avenues for the development of new drug candidates specifically tuned to induce non-apoptotic cancer cell death in unconventional ways.

## Methods

### Chemicals and reagents.

CIL56 was synthesized as described^13^ or the kind gift of Mark Smith (Stanford University, Stanford, CA). N,N,N,N-tetrakis[(pyridin-2-yl)methyl]ethane-1,2-diamine (TPEN), zinc pyrithione, and ZX1 were the kind gifts of Monther Abu-Remaileh (Stanford University, Stanford, CA). Lipopolysaccharide was the kind gift of Michael Bassik (Stanford University, Stanford, CA). Tegavivint (BC2059, Cat# HY-109103) was obtained from MedChemExpress (Monmouth Junction, NJ) or synthesized by Pharmaron Inc. Tegavivint structure and purity (> 99%) were confirmed using liquid chromatography-mass spectrometry (m/z 589.15) and 1H NMR. Both sources yielded identical results. Pralidoxime (Cat# HY-B1200), perillartine (Cat# HY-N2084), quinoline-val-asp-difluorophenoxymethylketone (Q-VD-OPh, Cat# HY-12305), thapsigargin (Cat# HY-13433), VPS34-IN-1 (Cat# HY-12795), VPS34-IN-2 (Cat# HY-12473),LGK974 (Cat# HY-17545), and DC661 (Cat# HY-111621 were obtained from MedChemExpress.. FIN56 was synthesized as described^13^. Fluvoxamine (Cat# B1205) was obtained from ApexBio (Houston, TX). HI-6 (asoxime chloride, Cat# SML0224), 2-butanone oxime (Cat# 332828), trimedoxime bromide (1,1’-trimethylenebis(4-hydroxyiminomethyl)pyridinium bromide, TMB-4, Cat# S458058), obidoxime chloride (Cat# 74593), 5-(tetradecyloxy)-2-furoic acid (TOFA, Cat# T6575), ferrostatin-1 (Fer-1, Cat# SML0583), arachidic acid (C20:0, Cat# A3631), menadione (Cat# M5625), menadione sodium bisulfite (Cat# M2518), 2-bromohexadecanoic acid (2-bromopalmitate, Cat# 21604), hydroxylamine hydrochloride (Cat# 379921), sodium fluoride (Cat# S6776), sodium dodecyl sulfate (Cat# L3771), protease inhibitor cocktail (Cat# P8340), and lithium chloride (Cat# L9650) were obtained from Sigma-Aldrich (St. Louis, MO). 3,4-dihydronapthalen-1(2H)-one oxime (Cat# AB3671) was obtained from AstaTech (Bristol, PA). Erastin2 (Cat# 27087), palmitic acid (Cat# 10006627), stearic acid (C18:0, Cat# 10011298), docosanoic acid (C22:0, Cat# 9000338), Δ2-*trans*-hexadecenoic acid (T2HDA, Cat# 11132), triacsin C (Cat# 10007448), Δ2-cis-hexadecenoic acid (C2HDA, Cat# 11133), Δ2-trans eicosenoic acid (T2EA, Cat# 10007622), DL-threo-PDMP (hydrochloride) (DL-PDMP, Cat# 10005276), and D-NMAPPD (Cat# 10006305), vacquinol-1 (Cat# 16321), miltefosine (Cat# 63280), edelfosine (Cat# 60912), and perifosine (Cat# 10008112) were obtained from Cayman Chemical Company (Ann Arbor, MI). 1*S*,3*R*-RSL3 (RSL3, Cat# S8155), IWR-1-endo (Cat# S7086), phorbol 12-myristate 13-acetate (PMA, Cat# S7791), MCC950 (Cat# S8930), elesclomol (Cat# S1052), zinc pyrithione (Cat# S4075), and JTC-801 (Cat# S2722), MOMIPP (Cat# E2933), and SAR405 (Cat# S7682) were obtained from Selleck Chemicals (Houston, TX). Bortezomib (Cat# NC0587961) was obtained from Thermo Fisher Scientific (Waltham, MA). Δ2-*trans*-octadecenoic acid (T2ODA, Cat# A1447265) was obtained from Ambeed Inc. (Arlington Heights, IL). Nigericin (Cat# tlrl-nig) was obtained from InvivoGen (San Diego, CA). CIL41 (Cat# Amb6320174) was obtained from Ambinter by Greenpharma (Orléans, FR). CIL70 (CAS# 337474–02-9) was obtained from LabNetwork Inc. (Chicago, IL). GNS561 (Cat# T62882) was obtained from TargetMol Chemicals Inc. (Boston, MA). Most small molecules were dissolved in DMSO. Pralidoxime, asoxime chloride, 2-butanone oxime, obidoxime chloride, hydroxylamine hydrochloride, sodium fluoride, sodium dodecyl sulfate, purified Wnt3a (Cat# 5036-WN-010, R&D Systems, Minneapolis, MN), LPS, MSB, edelfosine, miltefosine, and perifosine were dissolved in water. 2-bromopalmitate, palmitic acid, stearic acid, Δ2-trans-hexadecenoic acid, Δ2-cis-hexadecenoic acid, Δ2-trans-octadecenoic acid, Δ2-trans-eicosenoic, VPS-34-IN-2, and nigericin were dissolved in ethanol. All compounds were aliquoted and stored at −20°C until utilized for experiments. Lithium chloride was dissolved in BPS’ proprietary growth medium (Cat# 79531, BPS Bioscience, San Diego, CA). The one-step luciferase assay reagents were obtained from BPS Bioscience (Cat# 60690, San Diego, CA). Paraformaldehyde (PFA) was obtained from VWR (Cat# 100503–917, Radnor, PA).

### Cell lines and culture conditions.

SW 872 (white, male, Cat# HTB-92), SW 982 (white, female, Cat# HTB-93), SV40 *Bax*^*+/+*^ *Bak1*^*+/+*^ control MEFs (Cat# CRL-2907), SV40 *Bax*^−/−^ *Bak1*^−/−^ double knockout MEFs (Cat# CRL-2913), HPAF-II (white, male, Cat# CRL-1997), Capan-2 (white, male, Cat# HTB-80), Panc-1 (white, male, Cat# CRL-1469) and THP-1 (Asian, male, Cat# TIB-202) cell lines were obtained from ATCC. HT-1080^N^, A549^N^, and T98G^N^ cell lines were previously described^24, 25^. HT-1080^N^ Control and *ZDHHC5*^*KO1/2*^ cell lines were described previously^14^. HEK 293 Control and *ACSL3*^*KO1/2*^ cells were described previously^47^. The HEK 293 TCF/LEF luciferase reporter cell line (female, Cat# 60501) was obtained from BPS Bioscience (San Diego, CA). H460^C^ cells (male) expressing nuclear-localized mCherry^32^ were the kind gift of James Olzmann (University of California Berkeley). SW 872^N^, HPAF-II^N^, HAP-1^N^, and Panc-1^N^ cell lines were generated by transducing their respective parental cell lines with lentivirus directing the expression of nuclear-localized mKate2 protein. Clonal HT-1080^N^ Control and *TECR*^*KO1/2*^, SW 872^N^ Control and *TECR*^*KO1/2*^ and HAP1^N^ Control and *TECR*^*KO1/2*^ were generated using CRISPR/Cas9 technology (see below).

Upon receipt or generation, all cell lines were expanded, aliquoted, and frozen to ensure a low passage number for all experiments. All cell lines were maintained in humidified Forma^™^ Steri-Cycle^™^ CO2 incubators (184 L, Cat# 381, Thermo Fisher Scientific, Waltham, MA) at 37°C and 5% CO_2_. HT-1080 and T98G cells and derivatives thereof were maintained in Dulbecco’s Modification of Eagle’s Medium (DMEM, Cat# 10–013-CV, Corning Inc., Corning, NY) supplemented with 10% fetal bovine serum (FBS, Cat# 26400044, Thermo Fisher Scientific, Waltham, MA), 50 U/mL penicillin (Cat# 15070–063, Life Technologies, San Francisco, CA), 50 μg/mL streptomycin (Cat# 15070–063, Life Technologies, San Francisco, CA), and 1X MEM non-essential amino acids (Cat# 11140–050, Life Technologies, San Francisco, CA). SW 872 and SW 982 cells and derivatives thereof were cultured in DMEM/Hams F-12 50/50 Mix medium (Cat# 10–092-CV, Corning Inc., Corning, NY) supplemented with 10% FBS, 50 U/mL penicillin, and 50 μg/mL streptomycin. SV40 MEFs were cultured in Iscove’s Modified Dulbecco’s Medium (IMDM, Cat# 12440053, Thermo Fisher Scientific, Waltham, MA) supplemented with 10% FBS, 50 U/mL penicillin, and 50 μg/mL streptomycin. H460^C^ and THP-1 cells were cultured in RPMI-1640 medium (Cat# SH30027FS, Fisher Scientific, Hampton, NH) supplemented with 10% FBS (the FBS was heat-inactivated for THP-1 cells only), 50 U/mL penicillin, and 50 μg/mL streptomycin. HPAF-II cells were cultured in Eagle’s Minimum Essential Medium (EMEM, Cat# M4655, Sigma-Aldrich, St. Louis, MO) supplemented with 10% FBS, 50 U/mL penicillin, and 50 μg/mL streptomycin. Capan-2 cells were cultured in McCoy’s 5A medium (Cat# MT10050CV, Fisher Scientific, Hampton, NH) supplemented with 10% FBS, 50 U/mL penicillin, and 50 μg/mL streptomycin. HEK 293, A549, HAP1, and Panc-1 cells and derivatives thereof were cultured in DMEM supplemented with 10% FBS, 50 U/mL penicillin, and 50 μg/mL streptomycin. HEK 293 TCF/LEF reporter cells were thawed in BPS proprietary thaw medium (Cat# 60187, BPS Bioscience, San Diego, CA) and maintained in BPS proprietary growth medium (Cat# 79531, BPS Bioscience, San Diego, CA).

### Generation of mKate2 ^+^ stable cell lines.

HT-1080 (control, *TECR*^*KO1*^, and *TECR*^*KO2*^), HPAF-II, SW 872 (control, *TECR*^*KO1*^, and *TECR*^*KO2*^), HAP-1 (control, *TECR*^*KO1*^, and *TECR*^*KO2*^), and Panc-1 cell lines were engineered to express nuclear-localized mKate2^+^, a live cell marker. Cell sensitivity to puromycin (used in selection) was first determined. Cells were plated in a 96-well plate (Cat# 3598, Corning Inc., Corning, NY) at a density of 5,000 cells per well. The following day, cells were treated with puromycin in a 2-fold 11-point dose-response series (high dose = 16 μg/mL) in the presence of SYTOX Green (20 nM). Two days later, cell death was quantified using an IncuCyte (see below), and the minimum lethal concentration of puromycin was identified (1 μg/mL for all cell lines).

To generate stable cell lines, cells were plated in two wells of a 12-well plate (Cat# CLS3513, Sigma-Aldrich, St. Louis, MO) at a density of 30,000 cells per well. The next day, the medium was replaced with medium containing polybrene (8 μg/mL, Cat# H9268, Sigma-Aldrich, St. Louis, MO) and IncuCyte NucLight Red Lentivirus Reagent (5 μL/reaction, EF-1α, Puro, Cat# 4625, Essen BioScience, Ann Arbor, MI). The next day, the medium was replaced with medium containing the minimum lethal concentration of puromycin previously identified, and cells were incubated for at least two days to eliminate uninfected cells. At this point, the medium was replaced, and the cell line was expanded, aliquoted, and frozen down until needed to ensure a low passage number at the start of experimentation.

### Quantification of cell death.

Cell death was quantified primarily using the scalable time-lapse of cell death kinetics (STACK) method^25, 71^. In most experiments, cells that stably express nuclear-localized mKate2 were used. Cells were cultured in medium containing SYTOX Green to mark dead cells (20 nM, Cat# S7020, Life Technologies). Live-cell images were acquired using the 10x objective at regular intervals using an IncuCyte ZOOM (Cat# 4459) or an IncuCyte S3 (Cat# 4647) (Essen Biosciences, Ann Arbor, MI), and counts of red and green objects were quantified using the IncuCyte Software v2022b live cell analysis software and an automated analysis job. The following parameters were used for quantification of red (mKate2^+^) objects (i.e., live cells): adaptive parameters, threshold adjustment 5 GCU, edge split on, edge sensitivity - 2, filter area minimum 40 μm^2^, filter area maximum 8,100 μm^2^. The following parameters were used for quantification of green (SYTOX Green^+^) objects (i.e., dead cells): adaptive parameters, threshold adjustment 10 GCU, edge split on, edge sensitivity - 7, filter area minimum 40 μm^2^, filter area maximum 750 μm^2^. The lethal fraction score was calculated from counts of red and green objects as previously described^25, 71^. In some experiments, cell death was quantified by counting SG^+^ objects only. Here, SG^+^ counts were normalized to cell confluence at the start of the experiment. The following parameters were used to determine well confluence: segmentation adjustment 0.8, hole fill 50 μm^2^, filter area minimum 300 μm^2^. The following parameters were utilized to quantify SG^+^ objects: adaptive parameters, threshold adjustment 10 GCU, edge split on, edge sensitivity - 10, filter area minimum 5 μm^2^, and filter area maximum 800 μm^2^. For time course experiments, the maximum SG^+^ objects count throughout a time course were used, reflecting that long-dead cells that disintegrate and lose SG^+^ signals^25, 71^.

### High-resolution time-lapse imaging of compound-treated cells.

The day before the start of the experiment, 380,000 HT-1080^N^ Control or *TECR*^*KO1/2*^ cells per well were seeded into 6-well plates. The next day, the medium was replaced with medium containing SG (20 nM) and the appropriate lethal compounds, and imaging was performed using an Incucyte S3 every 10 min for 24 h using the 20x objective. SG^+^ and mKate2^+^ objects, and mKate2^+^area, were quantified using IncuCyte Software v2022b. For cell confluence, the following parameters were used: AI confluence segmentation, hole fill 50 μm^2^, adjust size (pixels) 0, and filter area minimum 300 μm^2^. For SG^+^ objects, the following parameters were used: adaptive segmentation, threshold adjustment 10 GCU, edge split on, edge sensitivity - 10, hole fill 0 μm^2^, filter area minimum 40 μm^2^, and filter area maximum 1500 μm^2^. For mKate2^+^ objects, the following parameters were used: adaptive segmentation, threshold adjustment 3 RCU, edge split on, edge sensitivity - 50, hole fill 10 μm^2^, filter area minimum 40 μm^2^, and filter area maximum 8100 μm^2^.

### Pyroptosis induction.

4 × 10^6^ THP-1 cells were differentiated in a 10 cm tissue culture dish by adding PMA to a concentration of 100 nM in 10 mL of medium. The cells were allowed to differentiate and adhere for 72 h. Then, cell adhesion was confirmed by examining the cells under a microscope. The cells were washed with 5 mL of HBSS with no magnesium and chloride (Fisher Scientific, Cat# 14-175-079). Then, 5 mL of pre-warmed Accutase (Thermo Fisher Scientific, Cat# 00-4555-56) was added. The cells were incubated at 37°C for 15 min and agitated every 5 min. After 15 min, the cells were collected into a 15 mL conical tube. Remaining adherent cells were lifted using a cell scraper (Cat# 07-200-364, Fisher Scientific, Hampton, NH). The cells were pelleted by centrifugation for 5 min at 500 × g at RT. The cells were resuspended in 1 mL of medium and counted. For cell death experiments, cells were diluted to a concentration of 25,000 cells/mL in medium containing 100 nM PMA and plated at a density of 5,000 cells per well per 200 μL in a 96-well plate or were diluted to a concentration of 50,000 cells/mL and plated at a density of 1,500 cells per well per 30 μL in a 384-well plate. For immunoblotting, the cells were diluted to a concentration of 190,000 cells/mL in medium containing 100 nM PMA and plated at a density of 380,000 cells per well per 2 mL in a 6-well plate. The following day, the cells were stimulated with LPS (gift of the Bassik Lab, Stanford University, Stanford, CA). at a concentration of 10 ng/mL. Four hours later, cells were treated with various lethal stimuli. Lethal fractions were computed using STACK (described above) or protein lysates were collected for western blotting. Nigericin (Cat# tlrl-nig, InvivoGen, San Diego, CA) was used as a positive control pyroptosis inducer, and MCC950 (Cat# S8930, Selleck Chemicals, Houston, TX) was used as a control pyroptosis inhibitor.

### Spheroid generation and analysis.

Cells were seeded in an ultra-low attachment 96-well plate (Cat# 6055330, Perkin Elmer, Waltham, MA) at a density of 10,000 cells per 100 μL per well. Three wells were seeded with just medium to be used as controls for background luminescence signal later. The outside wells of the plate were left empty since these wells are most susceptible to evaporation. Cells were incubated for 72 h to allow for spheroid formation (confirmed by IncuCyte imaging). Then, treatments were prepared at 2x in 2x SYTOX Green (40 nM for 20 nM final concentration) medium. 100 μL of each treatment was added to the appropriate wells containing 100 μL of media plus a spheroid for a final concentration of 1x compounds and 1x SYTOX Green in 200 μL total. After 24 h of treatment, 10 mL of CellTiter-Glo 3D viability assay reagent (Cat# G9681, Promega, Madison, WI) was placed at 4°C to thaw overnight. After 48 h of treatment, cells were imaged, and then viability was measured using the CellTiter-Glo 3D viability assay. 30 min before the measurement, the CellTiter-Glo reagent was placed in a 22°C water bath to equilibrate it to RT, and the spheroid plate was placed at RT. After the 30 min, 100 μL of medium was removed from each well of the plate using a multi-channel pipet, being sure not to aspirate the spheroids. 100 μL of CellTiter-Glo reagent was added to each well. The plate was covered in foil and shaken for 5 min on an orbital shaker at RT. Luminescence was measured using a Cytation3 multimode reader (Agilent). Average background luminescence (measured from wells lacking cells) was subtracted out from each experimental value, and cell viability was normalized within each cell line to the negative control treatment condition (e.g., DMSO only).

### Mouse xenograft experiments.

All animal experiments were approved by the Stanford Laboratory Animal Care committee (APLAC, protocol number 33882). The researcher performing these experiments, including generating the xenografts, administering the compounds and measuring tumor sizes and mouse masses, was blinded to the identity of both the cell lines and drug treatments. Forty nude mice (Strain #002019) were purchased from the Jackson Laboratory. Mice were allowed to acclimate to the new environment for two weeks before experimentation. Control and *TECR*^*KO2*^ HT-1080^N^ cells were expanded in two T175 flasks and allowed to grow until full confluence was reached. The day before tumor engraftment, matrigel (Corning #354230) was thawed at 4°C. On the day of tumor engraftment, each cell line was counted. 40 million cells per cell line were pelleted by centrifugation at 500 × *g* for 5 min. For each cell line, the cell pellet was resuspended in 2 mL of 1:1 matrigel:HT-1080 medium (described above) mixture for a final concentration of 1 million cells per 50 μL. 50 μL of cell suspension was injected into the thigh muscle of 20 nude mice per cell line using a 1 mL BD Luer-Lok^™^ syringe with BD Eclipse^™^ safety 27 G × 1/2 in (BD #305789). After tumors reached 0.7 cm in minimum diameter, treatments were initiated. To prepare the drug treatments, polyethylene glycol 12-hydroxystearate (Kolliphor solutol HS-15, Cat# HY-Y1893, MedChem Express, Monmouth Junction, NJ) was thawed in a 42°C water bath, and sterile PBS was warmed at 37°C for 30 min before drug preparation. 2.72 mL of a 1:1 mixture of ethanol:solutol HS-15 was made by mixing 1.36 mL of ethanol with 1.36 mL of solutol HS-15 and vortexing. This mixture was split into two tubes of 1.36 mL. To one tube, 100 mg of tegavivint was added. The mixtures were vortexed and sonicated until the tegavivint was fully dissolved. Then, 6.64 mL of sterile pre-warmed PBS was added to each tube for a final concentration of 8.5% solutol HS-15 (as reported previously^17^) and 12.5 mg/mL of tegavivint. Tegavivint or vehicle treatment were administered to the appropriate mice twice per week via intraperitoneal injection. Tumor volumes were recorded every other day and mouse masses were recorded twice weekly. Tumor volumes and mouse masses were recorded regularly. Once tumors tripled in size, or they reached 1.7 cm in maximum diameter, mice were sacrificed.

### Molecular biology.

Myc-DDK-tagged TECR (transcript 1) plasmid was purchased from OriGene Technologies (Cat# RC202503, Rockville, MD). Plasmid DNA was reconstituted in 100 μL of sterile nuclease free water for a final concentration of 100 ng/μL, and 1 μL of this solution was used to transform 50 μL of subcloning efficiency DH5α bacteria (Cat# 18265017, Thermo Fisher Scientific, Waltham, MA). Bacteria were then incubated on ice for 30 min followed by a 30 s heat shock in a 42°C water bath. Then, the bacteria were transferred back to ice and allowed to recover for 5 min. 950 μL of room temperature (RT) SOC outgrowth medium (Cat# B9020S, New England Biolabs, Ipswich, MA) was added to the bacteria. The tube was then shaken at 150 rpm for 1 h at 37°C. While the tube was shaking, LB agar plates with appropriate antibiotics were allowed to equilibrate to RT. 100 μL of the shaken bacterial mixture was added to the appropriate plates and spread with a sterile inoculating loop. pSpCas9(BB)-2A-GFP plasmid was purchased from Addgene (Cat# 48138, Watertown, MA) as a bacterial stab. A sterile inoculating loop was inserted into the stab and then streaked onto an LB agar plate containing appropriate antibiotic. After 5 min, plates were transferred to a 37°C incubator overnight. The next day, two isolated colonies with no satellites were isolated from each plate and transferred into 5 mL of LB broth with appropriate antibiotic and placed into a 37°C shaker to grow overnight. The next day, bacterial glycerol stocks were made with 900 μL bacteria suspension and 600 μL 50% glycerol in water. Then, bacteria were pelleted by centrifuging the suspensions at 4000 × *g* for 15 min at 4°C. Minipreps were prepared using the QIAprep Spin Miniprep Kit (Cat# 27115, Qiagen, Hilden, Germany). DNA concentration was measured using a Nanodrop 2000 spectrophotometer (Cat# ND2000, Fisher Scientific, Hampton, NH), and samples were sequenced by Sequetech Corporation (Mountain View, CA) or Elim Biopharmaceuticals, Inc. (Hayward, CA). An inoculating loop was dipped into bacteria glycerol stocks and subsequently dipped a 50 mL culture of LB with appropriate antibiotic. This mixture was shaken overnight at 37°C. The next day, the bacteria were pelleted and plasmid DNA was isolated using a miraprep^72^ as previously described using the QIAprep Spin Miniprep Kit (Cat# 27104, Qiagen, Hilden, Germany). The P182L mutation was created in the TECR plasmid using the New England Biolabs site-directed mutagenesis kit (Cat# E0554S, Ipswich, Massachusetts) by changing the proline codon (CCT) to a leucine codon (CTT). The forward primer used was 5’-ATCAATCACCtTCTCTACACTCC-3’ (with the altered nucleotide in lowercase), and the reverse primer was 5’-GTAATAGGCCATCCACGC-3’. The final plasmid was sequenced to confirm the presence of the appropriate mutation.

### Genome engineering.

CRISPR sgRNA sequences were chosen out of the top results from ChopChop (chopchop.cbu.uib.no/) and Synthego (synthego.com) and synthesized by Elim Biopharma, Inc. (Hayward, CA). CRISPR oligos were designed and cloned into the 100 ng pSpCas9_BB_2A_GFP using the selected sgRNA sequences and as previously described^73^. For the *TECR* CRISPR process, the forward oligo was 5’-CACCGTGATCTCCGCAATGGTGGCG-3’, and the reverse oligo was 5’-AAACCGCCACCATTGCGGAGATCAC-3’. Oligos were annealed together by mixing 1 μL of 100 μL of each primer with 8 μL ddH_2_O. The mixture was heated to 95°C then cooled to 25°C at a rate of 2.5°C every min in an Applied Biosystems ProFlex 96-well PCR System (Cat# 4484075, Waltham, MA). The resulting annealed complex was diluted at a ratio of 1:200 in nuclease-free water. The overhangs from the annealed oligo duplex allow for insertion into the pSpCas9_BB_2A_GFP plasmid upon linearization with the *Bpi*I restriction enzyme. The following reagents were mixed for cloning of the annealed oligo duplex into the pSpCas9_BB_2A_GFP plasmid: 100 ng pSpCas9_BB_2A_GFP (Cat# 48138, Addgene, Watertown, MA), 2 μL 1:200 diluted annealed oligo duplex, 2 μL of 10x FastDigest buffer (Cat# B64, Thermo Fisher Scienctific, Waltham, MA), 1 μL 10 mM DTT, 1 μL 10 mM ATP, 1 μL FastDigest *Bpi*I enzyme (Cat# D1014, Thermo Fisher Scientific, Waltham, MA), and 0.5 μL T4 DNA ligase enzyme (Cat# EL0014, Thermo Fisher Scientific, Waltham, MA) diluted to 20 μL in ddH_2_O. The mixtures were placed in a thermal cycler and run for 6 cycles (37°C for 5 min, 21°C for 5 min). The plasmids were transformed into bacteria and prepared as described above. Purified plasmids were transfected into cell lines as described below along with a water control well. Successful transfection was confirmed by imaging (Incucyte S3) to observe GFP positivity. Then, cells were lifted via trypsinization, resuspended in PBS, and passed through a 35 μm cell strainer (the cap of Cat# 352235, Corning Inc., Corning, NY) into a polypropylene round-bottom tube (Cat# 14-959-11A, Thermo Fisher Scientific, Waltham, MA). The suspended cells were single-cell sorted using a BD Aria Fusion, BD Aria II, or BD Influx Special Order using a 488 nm laser into two 96-well plates (Cat# 3598, Corning Inc., Corning, NY) per guide containing media with 20% FBS. Single-cell clones were allowed 2–4 weeks to grow and then transferred into larger flasks for validation of knockout via western blot and Sanger sequencing of the genomic region surrounding the target of the guides. A clone that went through the CRISPR process but did not show signs of being genetically disrupted via western blotting and sequencing was used as a control cell line.

### Transfection.

Cells were plated one day before transfection in a 12-well plate (Cat# CLS3513, Sigma-Aldrich, St. Louis, MO) at a seeding density of 90,000 cells/well. After 24 h, cells were transfected using Lipofectamine LTX with PLUS reagent (Cat# A12621, Thermo Fisher Scientific, Waltham, MA). An LTX master mix was made in Opti-MEM (Cat# 31985-062, Thermo Fisher Scientific, Waltham, MA) with enough LTX reagent for 2 μL per 50 μL per well plus overage. A DNA master mix (the DNA constructs used for overexpression are described in the molecular biology methods section) was made in Opti-MEM for each condition with enough DNA (or equivalent volume of water for control condition) and PLUS reagent for 0.5 μg DNA and 0.5 μL PLUS reagent per 50 μL per well plus overage. Each master mix was allowed to sit for 5 min. Then, the LTX mix was added to each DNA mix to a volume of 100 μL per reaction. Right after the mixtures were made, the cells were switched into pre-warmed medium containing no penicillin or streptomycin and kept in the incubator until transfection. After the 30 min elapsed, 100 μL of the appropriate mixtures was added dropwise to the appropriate wells. 6 h after transfection, the medium was replaced with regular growth medium with penicillin and streptomycin. 24 h after transfection, appropriate cell death or other treatments were added to the cells and death kinetics were measured using STACK.

### Immunoblotting.

For some experiments with no treatment conditions, such as whole cell lysate western blots of control and paired gene-disrupted cell lines, cells were seeded in 6-well plates (Cat# 3516, Corning Inc., Corning, NY) at a density of 380,000 cells per well and then harvested the next day. For most other experiments, cells were seeded in 6-well plates at a density of 190,000 cells per well and harvested two days after seeding. After 24 or 48 h, plates were placed on ice and washed twice with ice cold 1X phosphate buffered saline (PBS). 1X PBS was made by diluting 10X PBS, which was made from powder (Cat# 97062-338, VWR, Radnor, PA). Cells were harvested via scraping using a cell lifter (Cat# 07-200-364, Fisher Scientific, Hampton, NH) and pelleted in Eppendorf tubes (Cat# 1615-5500, USA Scientific, Ocala, FL) at 500 × *g* in a Sorvall Legend Micro 17 tabletop centrifuge (Cat# 75002431, Thermo Fisher Scientific, Waltham, MA) for 5 min. The supernatant was removed, and cell pellets were resuspended in radioimmunoprecipitation assay (RIPA) buffer with 0.1% sodium dodecyl sulfate (Cat# L3771, Sigma-Aldrich, St. Louis, MO) containing phosphatase inhibitor (5 mM sodium fluoride, Cat# S6776, Sigma-Aldrich, St. Louis, MO). and protease inhibitor cocktail (1:200 diluted P8340, Cat# P8340, Sigma-Aldrich, St. Louis, MO). The suspensions were sonicated at 60% amplitude for 10 cycles of 1 s on and 1 s off using a Fisherbrand Model 120 Sonic Dismembrator (Cat# FB120110, Fisher Scientific, Hampton, NH). Lysates were cleared by centrifuging at 18,213 × *g* and then transferring the supernatants to new Eppendorf tubes. Protein concentration was quantified using the Pierce bicinchoninic acid (BCA) Protein Assay Kit (Cat# 23225, Thermo Fisher Scientific, Waltham, MA) along with a 5-point curve of protein standards. Standardized samples were diluted with 4X Bolt LDS Sample Buffer (Cat# B0007, Thermo Fisher Scientific, Waltham, MA) and 10X Bolt Sample Reducing Agent (Cat# B0009, Thermo Fisher Scientific, Waltham, MA) to achieve 1X final concentration of each. Samples were loaded onto 10-well, 12-well, or 15-well Bolt 4–12% Bis-Tris Plus Gels (Cat# NW04120BOX, Cat# NW04122BOX, and Cat# NW04125BOX, Thermo Fisher Scientific, Waltham, MA), depending upon the number of conditions, along with one lane of 2.5 μL, 3 μL, or 3.5 μL (respectively) of Duo Chameleon pre-stained protein ladder (Cat# 928-60000, LI-COR Biosciences, Lincoln, NE). Gel electrophoresis was performed at 100 V for 1 h 50 min or until the dye front had just run off the gel. Then, proteins were transferred to nitrocellulose membranes using an iBlot2 (Cat# IB21001, Fisher Scientific, Hampton, NH) and the P0 protocol (20 V for 1 min, 23 V for 4 min, and 25 V for remainder). Membranes were blocked with Intercept blocking buffer (Cat# 927-70001, LI-COR Biosciences, Lincoln, NE). Primary antibodies were diluted in 3 mL of blocking buffer and added to hybridization bags containing the membranes and incubated overnight at 4°C. The following primary antibodies and concentrations were used: rabbit anti-cleaved caspase-3 (1:1000, Cat# 9664T, Cell Signaling Technologies, Danvers, MA), rabbit anti-GPX4 (1:1000, Cat# 125066, Abcam, Cambridge, UK), mouse anti-tubulin (1:2000, Cat# MS581P1, Fisher Scientific, Hampton, NH), rabbit anti-ZDHHC5 (1:1000, Cat# HPA014670, Sigma-Aldrich, St. Louis, MO), mouse anti-GOLGA7 (1:1000, Cat# H00051125-M01, Novus Biologicals, Centennial, CO), rabbit anti-TECR (1:1000, Cat# A305-515A, Thermo Fisher Scientific, Waltham, MA), mouse anti-β-catenin (1:1000, Cat# 610153, BD Biosciences, San Jose, CA), and rabbit anti-GAPDH (1:1000, Cat# 2118S, Cell Signaling Technologies). After overnight primary antibody incubation, membranes were washed 3 × 5 min in 1X tris-buffered saline with Tween (TBST). Membranes were then incubated at RT for one h in 15 mL secondary antibody mixture diluted in blocking buffer on an orbital shaker. The following secondary antibodies and concentrations were used: IRDye 680RD donkey anti-mouse IgG (1:15000, Cat# 926-68072, LI-COR Biosciences, Lincoln, NE), IRDye donkey anti-mouse IgG (1:15000, Cat# 926-32212, LI-COR Biosciences, Lincoln, NE), IRDye 680RD donkey anti-rabbit IgG (1:15000, Cat# 926-68023, LI-COR Biosciences, Lincoln, NE), and IRDye 800CW donkey anti-rabbit IgG (1:15000, Cat# 926-32213, LI-COR Biosciences, Lincoln, NE). Blots were rinsed with and stored in Milli-Q water prior to imaging. Membranes were imaged using a LI-COR Odyssey CLx in both the 700 nm and 800 nm channels. Images were adjusted and quantified using Image Studio software (LI-COR Biosciences).

### HAP1 CRISPR/Cas9 screen.

CRISPR/Cas9 screening in HAP1 cells was performed as described^74^. 100 million HAP1 cells stably expressing Cas9 were transduced with the TKOv3 lentiviral library (71,090 guide RNAs, total) at an M.O.I. of ~ 0.3. Then, after 24 h of recovery, cells were selected in puromycin (1 μg/mL) for a further 48 h. Cells were then split into three separate populations (Day 0), and on day 4, each population was subdivided into CIL56 (300 nM) treatment or DMSO arms and passaged every three days for a total of three treatment rounds. Cells were harvested on day 15, corresponding to ~ 17 population doublings for control-treated cell populations. Vehicle control and CIL56-treated samples were pelleted and prepared for next generation sequencing with data analysis conducted as previously described^75^. Cell death suppression scores (SumZ) were computed by comparing the effects on sgRNA representation in the final pools between DMSO control and CIL56-treated conditions.

### Gene silencing using short interfering RNA (siRNA).

The hs.Ri.HSD17B12.13.1 (Cat# 303800443) and hs.Ri.HSD17B12 13.3 (Cat# 303800446) siRNAs were obtained from Integrated DNA Technologies (Coralville, IA). Two nanomoles of siRNA powder was reconstituted in 100 μL of nuclease-free water for a concentration of 20 pM/μL. An AllStars negative control siRNA (Cat# 1027280) and an AllStars Hs death positive control siRNA (Cat# 1027298) were obtained from Qiagen (Germantown, MD). Five nmol of negative siRNA control and death positive siRNA control powder were each reconstituted in 250 μL for a final concentration of 20 μM. For all experiments, siRNAs were added to cells at the same time as cell seeding. siRNAs were diluted in Opti-MEM (Cat# 31985-062, Thermo Fisher Scientific, Waltham, MA) for a final concentration of 20 picomoles siRNA and 250 μL Opti-MEM per well in a 6-well plate (Cat# 3516, Corning Inc., Corning, NY) or 10 picomoles siRNA and 125 μL Opti-MEM per well in a 12-well plate (Cat# CLS3513, Sigma-Aldrich, St. Louis, MO), and the appropriate amount was added to each well. For experiments performed in 6-well plates, 2.5 μL Lipofectamine RNAiMAX reagent (Cat# 13778075, Life Technologies, Carlsbad, CA) in 250 μL Opti-MEM was used per well. For experiments performed in 12-well plates, 1.25 μL lipofectamine RNAiMAX in 125 μL Opti-MEM was used per well. The Lipofectamine/Opti-Mem mixtures were allowed to sit in the plate while cells were split and counted. HT-1080^N^ cells were added to the plates at a density of 180,000 cells per well for a 6-well plate and 90,000 cells per well for a 12-well plate. For each experiment, transfection efficiency was confirmed after 48 h by examination of the positive death control siRNA. Enough wells were plated for both RT-qPCR and cell death experiments. Cell harvests for qPCR were performed 48 h following transfection. Cell death experiments were initiated 48 h after transfection. Gene silencing was confirmed using RT-qPCR.

### Shotgun lipidomic analysis.

For each cell line, cells were plated in 10 cm plates at a density of 1,000,000 cells per 10 mL of medium per plate, with two plates per condition. The following day, the growth medium was replaced with medium containing DMSO or tegavivint (2.5 μM). After 5 h, plates were scraped using a cell lifter and transferred to Eppendorf tubes. Cells from each of the two plates per condition were consolidated into one tube. Cells were then pelleted at 500 × *g* for 5 min at RT. The supernatant was removed, and the pellets were washed with PBS and spun down again. The supernatants were removed, and pellets were frozen at −20° C and analyzed at the UCLA lipidomics core facility as described previously^49, 76^. Briefly, cells were transferred to extraction tubes with PBS. A modified Bligh and Dyer extraction^49^ as carried out on all samples. Prior to biphasic extraction, an internal standard mixture consisting of 70 lipid standards across 17 subclasses was added to each sample (AB Sciex Cat# 5040156, Avanti Cat# 330827, Avanti Cat# 330830, Avanti Cat# 330828 and Avanti Cat# 791642). Following two successive extractions, pooled organic layers were dried down in a Thermo SpeedVac SPD300DDA using ramp setting 4 at 35°C for 45 min with a total run time of 90 min. Lipid samples were then resuspended in 1:1 methanol/dichloromethane with 10 mM ammonium acetate and transferred to robovials (Thermo Cat# 10800107) for analysis. Samples were analyzed by direct infusion on a Sciex 5500 with Differential Mobility Device (DMS) with a targeted acquisition list consisting of 1,450 lipid species across 17 subclasses. The DMS was tuned with EquiSPLASH LIPIDOMIX (Avanti Cat# 330731). Data analysis was performed with an in-house data analysis workflow. Instrument settings, MRM lists, and analysis methods were described^77^. Quantitative values were normalized to cell counts. Any lipid species without data for more than one of four independent replicates was excluded.

### Neutral lipid staining and imaging.

One 18 mm #0 coverslip (MatTek Corporation, #NC1843886) was placed in each well of two 12-well plates. HT-1080^N^ Control and *TECR*^*KO1/2*^ cells were plated on top of the coverslips at a density of 140,000 cells per 2 mL per well. The next day, the medium was replaced with medium containing lipids at a concentration of 25 μM or a vehicle control. Six hours later, the cells were fixed by removing the media and replacing it with 4% PFA in PBS (500 μL per well). The cells were incubated for 20 min at RT. The fixed cells were washed 3 times with 1 mL PBS per well (the PFA and first wash were discarded into a specialized PFA waste container). A staining master mix was made by diluting BODIPY 493/503 and Hoescht into PBS for a final concentration of 1 μM BODIPY 493/503 and 1 μg/mL Hoescht. The cells were incubated for 30 min at 37°C. The cells were then washed twice with 1 mL PBS per well. The coverslips were mounted on microscope slides (Fisher Scientific, Cat# 12-550-143) with 5 μL ProLong Gold Antifade Mountant (ThermoFisher Scientific, Cat# P10144) and allowed to dry overnight. The next day, the slides were imaged using a BioTek Lionheart FX Automated Microscope (Agilent, Cat# 5994-4730EN) fitted with a 20X objective (Agilent, Cat# BT1220517). Hoescht was visualized using the DAPI 377/447 Rev M filter cube (Agilent, Cat# 1225100) connected to the 365 LED Rev J light source (Agilent, Cat# 1225007). BODIPY 493/503 was visualized using the GFP 469/525 Rev J filter cube (Agilent, Cat# 1225101) connected to the 465 LED Rev K light source (Agilent, Cat# 1225001). The number of lipid droplets per cell was quantified using a custom CellProfiler pipeline.

### RNA sequencing data.

RNA sequencing data for HT-1080 cells was reported previously^31^ and is available at https://datadryad.org/stash/dataset/doi:10.5061/dryad.jp43c.

### β-catenin reporter assays.

Wnt-3a ligand was dissolved in nuclease-free water at a concentration of 200 μg/mL, aliquoted, and stored at −80°C. A cell death assay was performed as described above to determine the death onset, lethal concentrations, and TOFA-suppressible concentrations of CIL56 and tegavivint for the HEK293 TCF/LEF luciferase reporter cell line (Cat# 60501, BPS Bioscience, San Diego, CA). Two concentrations of CIL56 and tegavivint were used: the maximum concentration that did not kill cells (sub-lethal concentration) and the minimum concentration that killed cells but could be suppressed by 1 μM TOFA (lethal concentration). For CIL56, these concentrations were 300 nM and 1 μM, respectively, and for tegavivint, these concentrations were 100 nM and 1 μM, respectively. IWR-1-endo (Cat# S7086, Selleck Chemicals, Houston, TX) was used at 1 μM. The assay was performed according to the manufacturer’s instructions. Specifically, cells were seeded in a white 96-well plate (Cat# 165306, Thermo Fisher Scientific) at 35,000 cells/60 μL medium per well. 20 μL of 40 mM lithium chloride (Cat# L9650, Sigma-Aldrich) dissolved in medium was added to each well to a final concentration of 10 mM. 20 μL of medium containing 10 mM lithium chloride and 5x concentration of IWR-1-endo, sub-lethal CIL56, sub-lethal tegavivint, or DMSO was added to the appropriate wells to a final concentration of 1x. For the lethal concentrations of CIL56 and tegavivint, cell death data were used to determine the time at which cells started to die. Then, for reporter assays, we used treatment times that corresponded to 90% of the death onset time for each compound (e.g., if cells started to die at 10 h, the assay would be performed at 9 h). Wnt-3a ligand was diluted to a concentration of 1.65 μg/mL in 10 mM lithium chloride dissolved in growth medium and added to the appropriate wells 20 h post-cell seeding. At 24 h post-cell seeding, the luciferase assay was performed on a BioTek Cytation3 multimode reader (Cat# BTCYT5FW, Agilent, Santa Clara, CA). After subtracting background signal (luminescence measured from DMSO + no Wnt3a-treated cells) from all samples, measurements were normalized to the DMSO + Wnt3a condition.

### Reverse transcription and quantitative polymerase chain reaction (RT-qPCR).

For experiments in which cells were harvested one day after seeding, cells were seeded in a 6-well plate at a density of 380,000 cells/well. For experiments in which cells were harvested two days after seeding, cells were seeded in a 6-well plate at a density of 190,000 cells/well. For experiments in which cells were harvested three days after seeding, cells were seeded in a 6-well plate at a density of 95,000 cells/well. At the end of the treatment period, cells were lifted from the plate using a cell scraper (Cat# 07-200-364, Fisher Scientific, Hampton, NH). Cells were spun down at 500 × *g* for 5 min in a Sorvall Legend Micro 17 tabletop centrifuge (Cat# 75002431, Thermo Fisher Scientific, Waltham, MA) and then washed with 1X PBS. The cells were spun down again at 500 × *g* for 5 min, and the supernatant was removed. The RNeasy Plus Mini Kit (Cat# 74134, Qiagen, Hilden, Germany) was used to extract RNA from the cell pellets. RNA concentrations were measured using a Nanodrop instrument. The number of reactions for each biological sample was calculated as follows: number of gene targets plus actin (control) × 3 technical. The total volume of cDNA needed for each biological sample was calculated as follows: 3 μL per reaction x number of reactions plus overage. Enough RNA from each biological sample was added to each PCR tube for 100 ng per reaction. In addition, the following reagents (and respective final concentrations) were added to each reaction: RT buffer (1X), MgCl_2_ (5.5 mM), deoxyNTPs mixture (0.5 mM), oligo dT (2.5 mM), random hexamers (2.5 mM), RNase inhibitor (0.5 U/L), and MultiScribe reverse transcriptase (3.125 U/L). All reagents are included in the TaqMan reverse transcription reagents product (Cat# N8080234, Thermo Fisher Scientific, Waltham, MA). The mixture was diluted to the appropriate total desired final cDNA volume, and the following protocol was run using a thermal cycler: Hold at 25°C for 10 min, hold at 48°C for 40 min, hold at 95°C for 5 min. Primers were designed using the NCBI primer design tool, and oligos were synthesized using Elim Biopharm’s oligo synthesis service. Primer sequences used were *HSD17B12* forward: 5’-TCGGAGAATGGGCAGTTGTCAC-3’, *HSD17B12* reverse: 5’-ACAACCTTCATTCCATGCTTTGC-3’, *CCND1* forward: 5’-ACCTGGATGCTGGAGGTCTG-3’, *CCND1* reverse: 5’-TCAGGGGGATGGTCTCCTTC-3’, *MYC* forward: 5’-CCTCTCAACGACAGCAGCTC-3’, *MYC* reverse: 5’-CTTCTTGTTCCTCCTCAGAGTCG-3’. For each gene target, the following master mix was made per reaction plus overage: 0.4 μL 5 μM forward + reverse primer mixture (final concentration 200 nM), 5 μL 2X Power SYBR Green PCR master mix (final concentration 1X, Cat# 4367659, Thermo Fisher Scientific, Waltham, MA), and 1.6 μL nuclease-free water. 25.2 μL of each master mix was pipetted into one PCR tube per biological sample. Then, 10.8 μL of each cDNA mixture was added to the appropriate PCR tubes and mixed by vortexing. Finally, 10 μL of each PCR tube mixture was added to each of 3 wells in a MicroAmp Fast Optical 96-well reaction plate with barcode (Cat# 4346906, Thermo Fisher Scientific, Waltham, MA). The plate was then sealed shut with a MicroAmp optical adhesive film (Cat# 4360954, Thermo Fisher Scientific, Waltham, MA). The following parameters were used to run the samples on a Applied Biosystems QuantStudio 3: instrument type: QuantStudio 3 System, block type: 96-well 0.1-mL block, experiment type: comparative C_T_ (ΔΔC_T_), chemistry: SYBR Green reagents, run mode: standard. The following protocol was run: 50°C for 2 min, 95°C for 10 min, 41 cycles of 95°C for 15 sec and 60°C for 1 min, and 95°C for 15 sec. Actin was used as a control for all experiments. C_T_ values were calculated using the built-in QuantStudio software. Average C_T_ values were calculated for each group from three technical replicates. ΔC_T_ values were calculated for each reaction by subtracting the C_T_ value for actin for the respective biological sample from the C_T_ value for that sample for each gene target. The Eq. 2^− ΔCT^ was used to calculate a final value for each sample. For each gene, relative expression was normalized to the negative control condition (DMSO).

### PRISM screening.

A PRISM screen^60, 61^ employing tegavivint was conducted at the Broad Institute. Briefly, cell line pools (20–25 cell lines per pool) were grown in RPMI and 10% serum (adherent) or 20% serum (non-adherent) were grown in 384-well plates. Initial seeding densities were 1,250 cells/well for adherent cells, and 2,000 cells/well for non-adherent cells. All cell pools were exposed to tegavivint for 5 d in an 8-point, 3-fold dose response, starting at a high dose of 10 μM. At this point, cells were lysed, and individual cell abundances were determined by analysis of unique expressed cell line barcodes using a Luminex platform, as described^60^. Mean fluorescent intensity values reflecting cell line abundance were used to compute compound area under the curve (AUC) values across all compound concentrations, as described^60^. Data for ML210 was obtained in the same way.

### Drug combination experiments.

For drug combination experiments, Control and *TECR*^KO^ HT-1080^N^ cells were plated in a 384-well plate at a concentration of 1,500 cells per 30 μL per well. The plate was briefly spun at 500 × g in a tabletop centrifuge to force the cells to the bottom of the plate. The following day, the cells were treated with selected drugs either individually or in combination. Specifically, a serial dilution of each drug was prepared in orthogonal directions on a drug dilution plate using SYTOX Green-containing medium. Then, the drug-containing medium was transferred to the plate containing the cells so that each cell line was treated with each combination of drug concentrations along with vehicle controls. The plates were scanned at 24 and 48 h to measure cell death induction and calculate lethal fraction scores. The rows and columns containing only one drug were used to calculate the expected lethal fractions of the drug combination wells. Specifically, the expected lethal fraction of a given drug combination was the sum of the lethal fractions for the wells with each individual drug treatment. If the individual lethal fractions summed to over 1, then the expected lethal fraction was set to 1. The difference between the expected and actual lethal fractions was plotted as a heatmap. Values of 0 indicated additivity (the lethal fraction of the drug combination conditions was equal to the sum of the individual lethal fractions). Values greater than zero indicated synergy (the lethal fraction of the drug combination was greater than that of the individual lethal fractions added together). Values less than zero indicated antagonism (the lethal fraction of the drug combination was less than that of the individual lethal fractions added together).

### Software, data processing and data display.

Gene dependency data were obtained from the Cancer Dependency Map Project at the Broad Institute (DepMap, 24Q2, https://www.depmap.org/portal, Broad Institute, Cambridge, MA), accessed on October 25, 2024. Cell death, qPCR, and western blot quantification data were analyzed using Microsoft Excel (Version 16.81, Microsoft, Redmond, WA) and visualized using GraphPad Prism 10 (Dotmatics, Boston, MA). IncuCyte data were obtained and exported using the IncuCyte 2022b Rev2 software (Sartorius, Göttingen, Germany). DNA sequences and plasmids were visualized using the Snapgene (Version 7.1.2, GSL Biotech LLC, San Diego, CA). Figures were generated using Adobe Illustrator (Version 28.1, Adobe, San Jose, CA).

### Statistical analysis.

A two-way ANOVA was conducted using Graphpad Prism 10 to test for statistically significant differences in xenograft tumor tripling time between genotypes (level 1) and treatment conditions (level 2). Tukey’s multiple comparisons test was used to determine statistical significance between the four conditions. A two-tailed, two-sample unequal variance (heteroscedastic) t-test was used to compare mouse body weights.

## Data Availability

Unprocessed immunoblots can be found in the **Extended Data Fig. 10**. DepMap data is publicly available (https://depmap.org/portal/). All other raw data will be made freely available by the senior author (S.J.D.).
